# Comparison of pre-processing methodologies for Illumina 450k methylation array data in familial analyses

**DOI:** 10.1186/s13148-016-0241-2

**Published:** 2016-07-16

**Authors:** Emma Cazaly, Russell Thomson, James R. Marthick, Adele F. Holloway, Jac Charlesworth, Joanne L. Dickinson

**Affiliations:** Menzies Institute for Medical Research, University of Tasmania, Private Bag 23, Medical Sciences Building 2, Hobart, TAS Australia; Centre for Research in Mathematics, School of Computing, Engineering and Mathematics, Western Sydney University, Parramatta Campus, Locked Bag 1797, Penrith, NSW 2751 Australia; School of Medicine, University of Tasmania, Medical Sciences Building 2, Hobart, TAS 7001 Australia

**Keywords:** Familial data, 450k, Array, Methylation, Pre-processing pipeline, Normalisation

## Abstract

**Background:**

Human methylome mapping in health and disease states has largely relied on Illumina Human Methylation 450k array (450k array) technology. Accompanying this has been the necessary evolution of analysis pipelines to facilitate data processing. The majority of these pipelines, however, cater for experimental designs where matched ‘controls’ or ‘normal’ samples are available. Experimental designs where no appropriate ‘reference’ exists remain challenging. Herein, we use data generated from our study of the inheritance of methylome profiles in families to evaluate the performance of eight normalisation pre-processing methods. Fifty individual samples representing four families were interrogated on five 450k array BeadChips. Eight normalisation methods were tested using qualitative and quantitative metrics, to assess efficacy and suitability.

**Results:**

Stratified quantile normalisation combined with ComBat were consistently found to be the most appropriate when assessed using density, MDS and cluster plots. This was supported quantitatively by ANOVA on the first principal component where the effect of batch dropped from *p* < 0.01 to *p* = 0.97 after stratified QN and ComBat. Median absolute differences between replicated samples were the lowest after stratified QN and ComBat as were the standard error measures on known imprinted regions. Biological information was preserved after normalisation as indicated by the maintenance of a significant association between a known mQTL and methylation (*p* = 1.05e-05).

**Conclusions:**

A strategy combining stratified QN with ComBat is appropriate for use in the analyses when no reference sample is available but preservation of biological variation is paramount. There is great potential for use of 450k array data to further our understanding of the methylome in a variety of similar settings. Such advances will be reliant on the determination of appropriate methodologies for processing these data such as established here.

**Electronic supplementary material:**

The online version of this article (doi:10.1186/s13148-016-0241-2) contains supplementary material, which is available to authorized users.

## Background

DNA methylation, the covalent addition of a methyl group to a cytosine base, usually in a cytosine-guanine pair (CpG), remains the most widely studied epigenetic modification in disease. While around 70 % of CpG dinucleotides are methylated in mammals, when clustered in groups or ‘islands’ (CGIs) they are generally unmethylated [1]. These islands occur often at promoter regions, where methylation has been traditionally associated with transcriptional repression [2]. Less extensively studied, but potentially more interesting, is the regulatory role of methylation at CpG shores and within gene bodies, as these regions have been found to be more variably methylated between tissue types and in cancer compared to normal tissue [3, 4].

Deepening the complexities surrounding the regulatory roles of CpG dinucleotides located in regions adjacent to promoters, ‘shores’ and gene bodies is the knowledge that sequence variation has a strong influence on methylation. Gertz et al. [5] examined methylation patterns in a three generation family and have estimated that genotype explains around 80 % of the variation in methylation. Methylation quantitative trait loci or meQTLs refer to sequence variants across the genome driving methylation patterns [6] and these have been mapped in a variety of different tissues and at different stages of development in various organisms [7–10]. Smith et al. [9] have compared sequence variants influencing methylation patterns across different human tissues and identified sets of meQTLs that are tissue specific but also others that are consistent across different tissue types and indeed across populations. Further, inherited genetic variants have been linked to methylation changes observed in disease. Shen et al. [11] have demonstrated that susceptibility SNPs at the *HNF1B* locus in ovarian cancer are associated with altered methylation and consequent expression of *HNF1B*. Also, it has been proposed that at least a proportion of unexplained Lynch syndrome cases are likely to be due to epigenetic silencing of mismatch repair genes. Indeed, it has been shown that the inheritance of the c.-27C>A germ-line variant in the 5′ UTR leads to epigenetic silencing *MLH1* in Lynch syndrome [12]. Thus, there is now considerable interest in mapping inherited methylation changes influencing disease susceptibility and disease course.

Genome-wide epigenetic studies have thus far largely focused on epigenetic alterations that occur in diseased tissues, where epigenetic changes across the genome are mapped through comparing ‘normal’ and affected tissues from the same individual. Indeed, epigenetic drugs, currently in clinical use, are designed to correct the epigenetic alterations acquired during disease development [13]. The assumption being that these acquired epigenetic alterations are driven by the disease process itself. More recently, it has been hypothesised that inherited genetic variation can drive epigenetic alterations and further that these contribute to disease susceptibility or disease course. To date, the large majority of genome-wide methylation studies and consequently the bioinformatic pipelines used to interpret these data have been designed to compare diseased with ‘normal’ tissue, in order to map epigenetic changes in the disease tissue itself. This analysis may screen out inherited epigenetic changes that are evident both in the normal tissue and the diseased tissue of the same affected individual. There remains a need to explore inter-individual variation of the epigenome and its contribution to disease. A powerful approach to examining the role of inherited variation drivers of epigenetic change is to examine large families where inheritance of variation driving epigenetic alterations can be tracked through generations.

A number of challenges exist in the analysis of genome-wide methylation mapping in samples and these include technical challenges dealing with batch effects and the underlying biochemistry employed by the array methods. This has necessitated the development of numerous pre-processing quality control methods to ensure reliable, high-quality data generation. As most studies examining epigenetic profiles have typically examined differences between two distinct groups (normal vs tumour tissue or case vs control), the majority of normalisation methods for the 450k array are designed for these types of data, frequently requiring two data groups to normalise negative and positive control probes or genomic regions. Such methods are incompatible with pedigree data, which lack a distinct second group for normalisation. In response to the absence of appropriate strategies, we have developed a pipeline for optimal normalisation and pre-processing of familial-based methylation array data.

## Methods

### DNA isolation and preparation

**Fig. 1:**
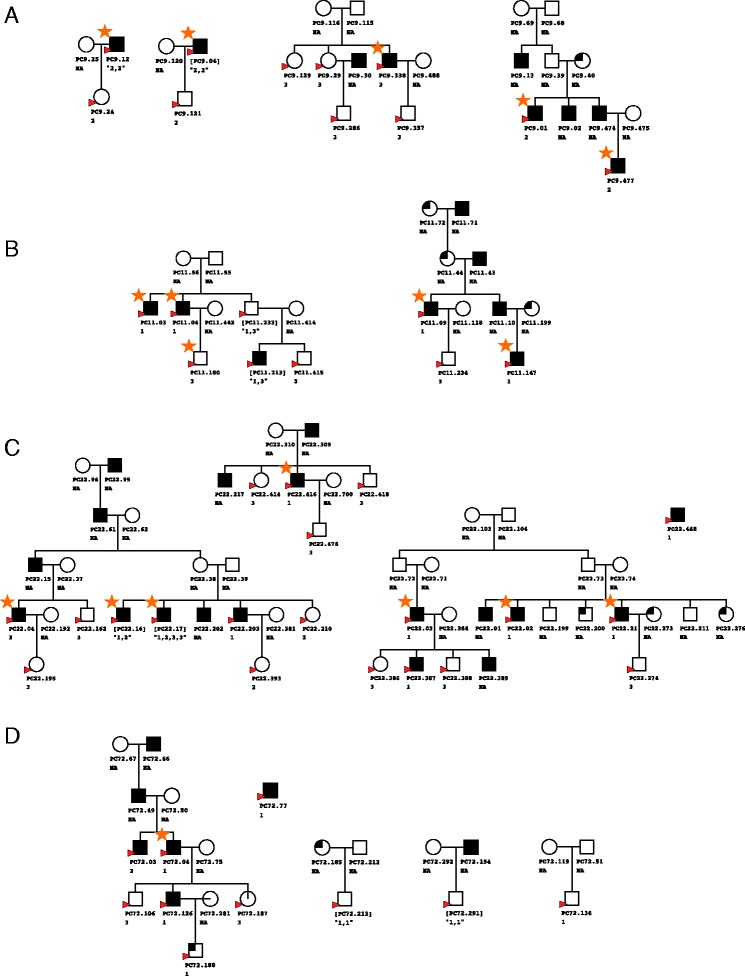
Selected pedigree clusters from four families from the Tasmanian Familial Prostate Cancer study. Four clusters were chosen from family 9 (**a**), two from family 11 (**b**), four from family 22 (**c**) and five from family 72 (**d**). *Circles* represent women and *squares* men, with individuals affected by prostate cancer filled in *black*, those unaffected unfilled and individuals affected by other cancers quarter filled. Samples interrogated on the 450k array are indicated by a *red arrow head*. Replicate samples are indicated by *square brackets* around the sample name, while the batch is indicated underneath the sample name. *Orange stars* indicate samples for which good-quality Omni2.5 genotype and 450k methylation data were available

Fifty peripheral blood samples were collected from individuals representing clusters of densely aggregated cases of affected men and close relatives from the Tasmanian Familial Prostate Cancer study. A diagrammatic representation of the family pedigrees is shown in Fig. 1, with disease status indicated. Individuals are of Caucasian descent, ranging in age from 23 to 89 years. See Additional file 1: Table S1 for more detailed information on clinical data and sample handling where available. DNA was extracted from whole blood using the Nucleon BACC3 (GE Healthcare) DNA extraction kit, following the manufacturer’s instructions. DNA was initially quantified on the Nanodrop 8000 (Thermo Scientific) and samples with a 260:280 ratio of less than 1.80 were further purified using the Zymo Clean & Concentrator (TM)-5 Kit. DNA was then quantified using a Qubit® Flourometer. One microgram of DNA was bisulphite converted using the EZ DNA Methylation-Gold (TM) kit (ZYmo Research), according to the manufacturer’s instructions. Bisulphite-converted DNA (400 ng) was then used for analysis of DNA methylation using the 450k array, according to the manufacturer’s instructions.

### Data extraction, pre-processing and initial quality control

IDAT files containing the raw intensity signals from red and green colour channels were generated using Illumina’s *iControl* software, with all further analysis carried out in the R environment [14]. A combination of three R packages, *minfi* [15], *methylumi* [16] and *ChAMP* [17], were used to load *IDAT* files into R and perform basic quality control. Different normalisation methods require the data to be in different formats which cannot be subsequently modified once loaded into *R*. As such, a number of different packages were used to load data, with the chosen package dependent on the normalisation method tested. *Methylumi* was used to read data into R in the correct format for quantile normalisation in the *lumi R* package. The *minfi* package provides a quality control report based on inbuilt control probes on the array (such as staining, hybridization, bisulfite conversion and negative controls) as well as the ability to exclude probes and samples based on probe signal intensity. Samples failing this initial quality control were excluded from further analysis. Replicate samples across batches were included on the beadchips to allow assessment of quality control and technical bias. Of the 50 unique samples and 8 replicates initially interrogated, 45 unique and 5 replicate samples passed quality control metrics and were used for further analysis. Following sample quality control, the recommended quality thresholds in ChAMP were employed to exclude poor quality probes, with a minimum detection *p* value of 0.05 in more than one sample removing 6740 probes and a bead count threshold of <3 in 5 % of samples removing a further 478 probes. To account for sex differences in methylation, driven particularly by dosage compensation by X-inactivation, probes on the sex chromosomes were removed prior to normalisation. While ChAMP includes this option as default when loading data, most packages require manual separation, normalisation and recombination of sex chromosomes or their complete manual removal. Thus, to permit appropriate comparison of normalisation methods, a homogenous set of loci across all packages was required; therefore, sex chromosomes were removed at this stage of analysis and not re-introduced.

### Normalisation

Eight normalisation techniques were applied to the whole dataset, as detailed in Table 1 with each method evaluating the same samples. The probe subset chosen for each analysis was selected following the instructions of each individual normalisation package, which had different requirements. This dictated whether normalisation methods were compatible and could be used in conjunction.

**Table 1:** Normalisation methods tested. The table includes a brief description of each method, the relevant R package and reference for further information

Normalisation method	Package	Reference
*Quantile normalisation* The distributions of probe intensities for different samples are made identical. Often used in microarray analysis.	lumi	[33]
*Stratified quantile normalisation* Probes are stratified by genomic region then quantile normalised with sex chromosomes normalised separately when male and female samples are present. No background correction, zeros removed by outlier function. Not recommended for cancer-normal comparisons or other groups with global differences.	minfi	[15]
*Beta-mixture quantile dilation (BMIQ)* Adjusts type II probes to type I distribution. Recommended for all datasets.	ChAMP	[27]
*Subset-quantile within array normalisation (SWAN)* A quantile distribution is created using a subset of probes, with subsetting based on the number of CpGs in the probe body. Separate subsets are created for type I and II probes. The remaining probes are then adjusted to the subsets.	minfi	[34]
*Functional normalisation (FunNorm)* Uses control probes to remove unwanted technical variation. Also diminishes batch effects in some datasets. Suitable for use in cancer-normal studies or where global methylation changes occur.	minfi	[29]
*Dasen* Background adjustment and between array normalisation are performed separately on type I and II probes.	wateRmelon	[20]
*Noob* Uses type I probe design to measure non-specific fluorescence in the opposite colour channel.	minfi	[35]
*Remove unwanted variation (RUV)* Previously used with microarray data to normalise via negative control genes. Requires distinct groups such as cancer-normal to normalise on.	RUVnormalize	[36]
Batch correction: *ComBat* Adjusts for known or unknown batches using an empirical Bayesian framework.	sva	[19]

Data are presented for each method except RUV, for which the results were not resolvable using the data generated in this study. These methods involve various degrees of type I and II probe scaling to account for underlying technical differences between the probe types, background and dye bias correction and initial between array batch correction. Depending on the normalisation method, data was either used in the red/green signal format (RGset), converted into methylated and unmethylated values (MethylSet) or converted to β values by the function *β* = *M*/(*M* + *U* + 100), where *M* is the methylated signal and *U* unmethylated. In some normalisation methods, the offset of 100 is included to regularise scores when both methylated and unmethylated values are very low. While the β value is more biologically intuitive (it ranges from 0 to 1 indicating the proportion of methylation at that site for the population of cells analysed), it suffers from severe heteroskedasticity at very high or low values [18]. Logit transforming to an *M* value removes this unequal variance. Thus wherever possible, calculations in this study have been performed on the *M* values and transformed back to β values if required for biological interpretation. Eight performance metrics were then used to compare methods and determine the optimal normalisation approach for familial datasets. Visual tools such as density and MDS plots and unsupervised hierarchical clustering were used to compare the various methods between all samples and particularly replicate samples. See Table 2 for a description of each metric.

**Table 2:** Qualitative and Quantitative metrics used to assess normalisation efficacy. The table includes a brief description of each metric and which figures describe the results for that method

	Method	Description	Figure
1	Density plot: all samples	Bimodal distribution of Beta values as methylated and unmethylated signals. Each sample is represented by a single line. A batch effect is indicated when samples performed in the same batch have a similar distribution.	Fig. 2a, c, e Additional file 5: Figure S4
	Density plot: three groups of replicate samples	Bimodal distribution of Beta values as methylated and unmethylated signals. Samples are coloured by replicate group. As each replicate group contain the same biological information, differences in sample distribution within groups indicate technical bias.	Additional file 3: Figure S2 (A, C, E)
	Density plot: probe I and II distribution	Bimodal distribution of Beta values as methylated and unmethylated signals separated by Infinium I and II probe types. Provides information about probe normalisation which is required for Infinium I and II signals to be combined in the same analysis.	Fig. 2b, d, f
2	MDS plot: all samples	Multidimensional scaling plots show a 2D projection of distances between samples. For these plots the 1000 most variable sites have been selected as they are the most biologically relevant for this type of analysis. Samples cluster by similarity and as such batch effects and familial clustering can be clearly discerned.	Fig. 3 Additional file 8: Figure S5
	MDS plot: three groups of replicate samples	1000 most variable sites are again selected, with samples coloured by replicate group. As each replicate group contains the same biological information, close within group clustering indicates minimal technical bias while distantly clustered replicate samples indicate heightened technical bias.	Additional file 3: Figure S2 (B, D, F)
3	ANOVA of the first principal component for MDS plots	Provides a quantitative value for MDS plots. A lower *p* value indicates the clustering is more significantly explained by batch. Ie. a larger *p* value after normalisation indicates a reduction in batch effect.	*p* values displayed on Fig. 3
4	Median absolute differences between replicate samples	For each replicate group the median *M* value (log of Beta values) across all probes was calculated and the absolute difference compared between replicate groups after various normalisation methods. A smaller absolute difference indicates improved normalisation as more technical bias is removed.	Additional file 6: Table S2
5	Imprinted regions: density plots	227 probes mapping known imprinted hemi-methylated regions can be used as a standard to measure changes in methylation levels after normalisation. Density plots have a single distribution peak since there is roughly 50 % methylation at these sites.	Additional file 4: Figure S3
	Differentially methylated region standard error (DMRSE)	The DMRSE measures how each sample varies from the expected 50 % methylation. Smaller error/deviation from 50 % indicates less technical bias.	Additional file 1: Table S1 Additional file 4: Figure S3 (A, C, E)
6	Cluster dendrogram	Another tool to measure clustering by sample similarity. Samples are labelled by batch with batch effects clearly seen before normalisation and diminished after. Red stars indicate replicate samples that are expected to cluster most closely.	Additional file 2: Figure S1
7	meQTL association	Association between methylation at cg17749961 and SNPs in a 2-Mb window. A significant association is maintained after normalisation and batch correction.	Additional file 5: Figure S4
8	Epigenome-wide methylation association with age	QQ plots depicting the association between epigenome-wide methylation and age. Plots are performed on raw, normalised and batch-corrected data.	Additional file 9: Figure S6

### Batch correction

Since an obvious batch effect remained after normalisation, the ComBat function from the *sva* package [19] was used to further remove technical bias introduced by interrogating samples on the 450k array in different batches.

### Genotype data

DNA from a subset of samples was extracted as described above and interrogated on Illumina’s HumanOmni2.5-8 Beadchip according to the manufacturer’s instructions. Quality control was performed with Illumina’s *GenomeStudio* Software.

### Statistical analysis

Eight methods, as described in Table 2, were used to compare the efficacy of the various normalisation methods. In addition to density and MDS plots, the ANOVA test and quantitative measures, mean absolute difference between replicates and the differentially methylated region standard error (DMRSE) measures were used. Additionally, two approaches were taken to test the underlying biological information was preserved between samples; namely, an association analysis between genotype and methylation at a previously identified meQTL and an epigenome-wide association analysis with age.

For a qualitative measure to examine effectiveness of between array normalisation, hierarchical cluster dendrograms were generated using all probes with the *hclust* function using the Euclidean distance between from the default R package, *stats*. Cluster dendrograms group samples by differences, with similar samples grouping together.

**Fig. 2:**
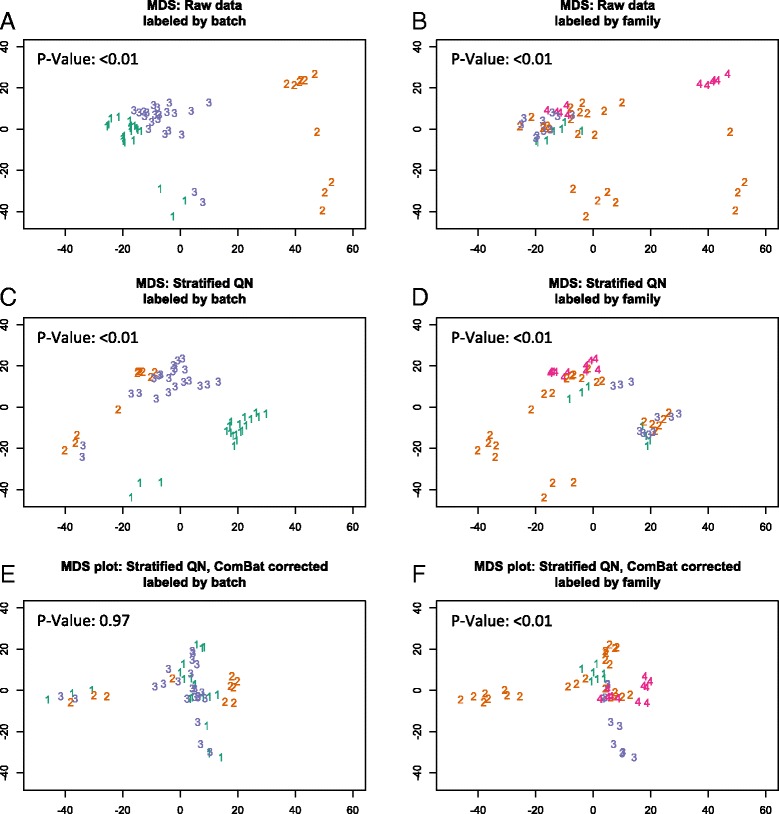
Multidimensional scaling plots of *M* values by batch and family. Multidimensional scaling plots for raw (**a**, **b**), stratified QN (**c**, **d**) and ComBat stratified QN (**e**, **f**) *M* values. For each plot, the 1000 most variable probes were selected. In **a**, **c** and **e**, *numbers* represent batches and are *coloured* accordingly, with clustering by batch clearly seen in **a**, to a lesser extent in **c** and removed in **e**. In **b**, **d** and **f**, *numbers* represent family groups and are *coloured* accordingly with the clearest clustering present in **f** after the batch effect has been removed. *p* values are from an ANOVA test for significance of batch (**a**, **c** and **e**) or family (**b**, **d** and **f**) on the first principal component from a PCA on the beta values

MDS plots were clustered by batch or family; then, analysis of variance was performed on the first principal component from a PCA on the 1000 most variable beta values using the *aov* and *prcomp* functions in the *stats* core R package. *p* values are displayed on the MDS plots in Fig. 2. A lower *p* value indicates that clustering is more significantly explained by batch or family, with a larger *p* value after normalisation indicating a reduction in technical bias.

Six replicate sample pairs were used to quantitatively assess the performance of the normalisation methods, as one sample from each pair was interrogated on a separate batch. The median absolute difference between each pair was calculated by first taking the absolute difference at each probe between the two replicates and then taking the median of the differences. A lower median difference indicates less technical bias, as the samples are biologically identical.

There are 227 known imprinted regions (iDMRs) on the 450k array, and these have previously been employed in analysis packages such as *wateRmelon* as a quality control metric [20]. These regions are expected to have allele-specific methylation and a β value of 0.5, and therefore deviation from this value can be examined as a standard error-type measure, denoted DMRSE in the *wateRmelon* package. The *dmrse_row* function was used to measure dispersion of methylation between samples for each normalisation method. A lower value indicates methylation values are more tightly aligned with expected methylation levels.

While evidence of clustering according to familial relationships following normalisation correction provides some confidence that biological integrity of the data is preserved, to further test the preservation of biologically relevant information, we examined detectable associations of known meQTLs in our data. Shoemaker and colleagues have previously identified 736 CpG sites to be associated with SNPs in *cis* [21]. Here, we examined cg17749961, one of the ten most significant hits reported by Shoemaker et al., in the 22 individuals, for whom both methylation and genotyping SNP data was available. Association analysis was performed between this probe site and SNPs located within a 2-Mb window adjacent to this site, using linear regression, and assuming an additive disease model. Relatedness was adjusted for by fitting a linear mixed model on the methylation of cg17749961 and a kinship matrix, determined by the identity-by-state function in the *GenABEL* R package [22]. The residuals from this model were then used as the outcome variable in the linear regression model with SNPs drawn from a 370 K Illumina array. Bonferroni correction was used to correct for multiple testing error.

To further demonstrate biological information is preserved after normalisation and batch correction, the association between age and epigenome-wide methylation was compared for raw data, stratified QN normalised data and ComBat-corrected stratified QN data. Linear regression models were fitted with age as the explanatory variable and methylation as the outcome variable, with −log10 *p* values of the models plotted against −log10 expected *p* values as QQ plots.

## Results

### Evaluation of normalisation methods to address technical bias

Data generated from whole genome methylation analysis employing array technology generates an output necessitating application of normalisation methods to correct for possible bias arising from within and between array variation. Herein eight different methodologies (Table 1) were examined and visual and quantitative metrics were employed to evaluate their comparative performance. High-quality methylation data was generated for 45 unique and five replicate samples from four families using the 450k array in three separate batches (see Fig. 1 for further details). A minimum of one sample in each of the three batches was replicated, providing five technical replicates in addition to the three unique samples on each batch, to permit generation of data from analysis of the same biological sample. In data lacking technical bias, replicate samples would be expected to generate the most similar methylation profiles, while methylation profiles generated from closely related individuals should also cluster tightly compared to distantly or unrelated individuals. However, if technical bias such as a batch effect has been introduced, this distorts the profiles and samples no longer cluster by biological similarity but instead the most evident grouping would be by batch.

**Fig. 3:**
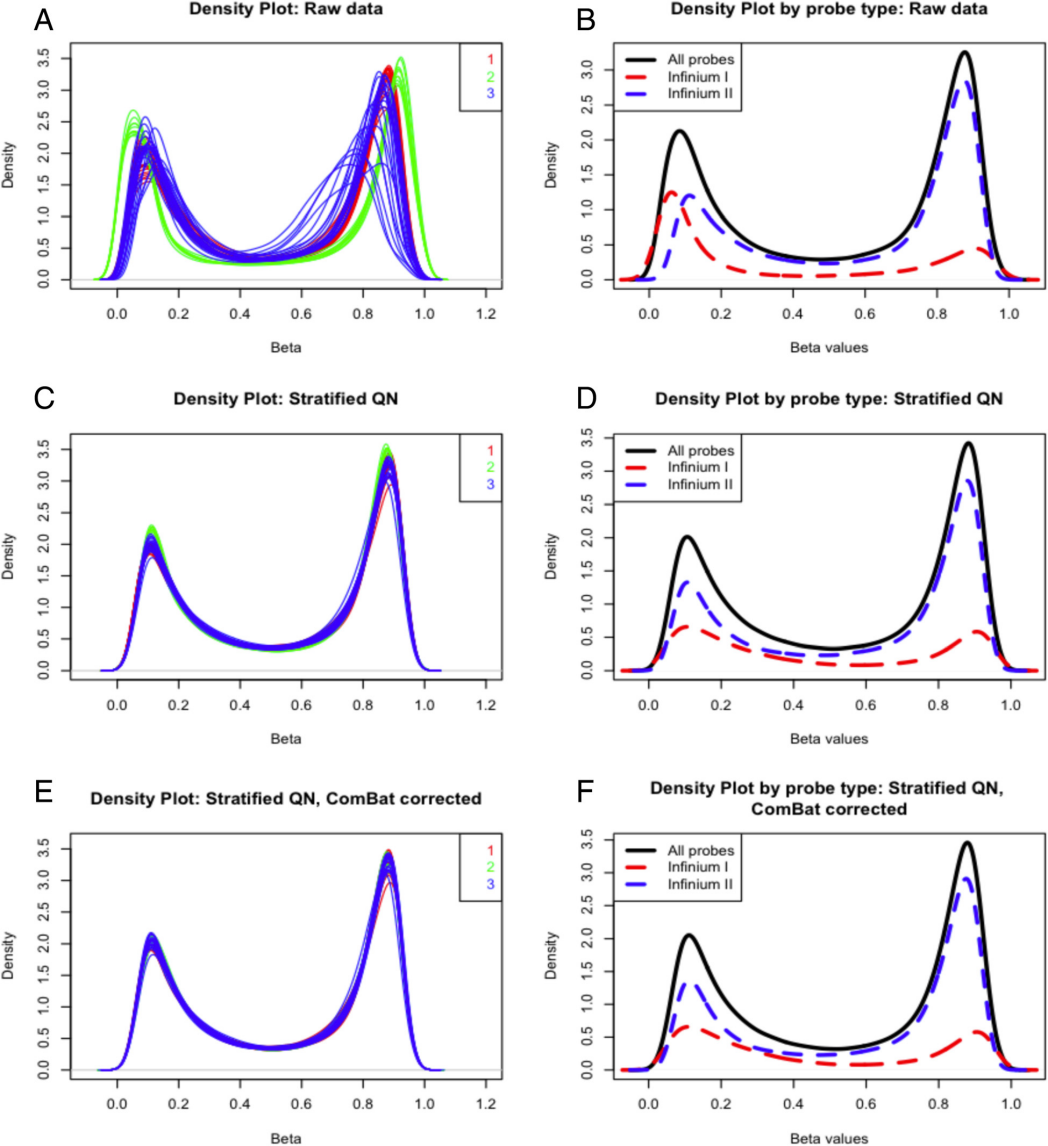
Density distribution of β values. Density plot and probe distribution of β values for raw pre-normalisation data (**a**, **b**), after stratified QN (**c**, **d**) and with stratified QN and ComBat batch correction (**e**, **f**). For density plots (**a**, **c**, **e**), a *single line* represents a sample, with samples *coloured* by batch. A clear batch effect is present in **a**, lessened in **c** and removed in **e**. For the probe distribution (**b**, **d**, **f**), one sample has been chosen with the *red dashed line* indicating type I probe distribution, the *blue dashed line* type II and the *solid black line* the combined probe distribution. The probe type distribution is also improved after normalisation, as types I and II are more closely aligned in **d** and **f** compared to **b**

Batch effect (between array variation) was examined and the density distribution plot (Fig. 3a) of the raw β values from all three batches reveals significant bias. The greatest contributor to batch effect was the date on which the BeadChips were processed, with bisulphite conversion performed on the same day as BeadChip processing. Employing a stratified QN (Fig. 3b) and/or ComBat normalisation (Fig. 3c) dramatically reduced this observed effect. For between array biases, Fig. 3 shows the density distribution of β values for raw data samples (A), after stratified QN (C) and after stratified QN combined with ComBat correction (E). This is particularly evident when comparing the β value density plots of three groups of replicate samples (Additional file 2: Figure S1A, C and E).

Stratified QN also performs best at removing within array biases as the distribution of probe I and II types become more uniform (Fig. 3b, d, f). This bias is driven by the differing biochemistry of the probes, with type I employing a single colour channel with a different bead for methylated and unmethylated DNA and type II containing one bead in two colour channels. The underlying biology targeted by each probe is confounded by this technical bias, as type I measures CpG-dense regions (such as islands) while type II can only tolerate three CpGs in the length of the probe. As such, type I interrogates a greater proportion of unmethylated to methylated DNA, while type II performs the opposite. Removing the probe bias is imperative for accurate comparisons between these probe types when pooling probe I and II data, which is necessary for accurate genome-wide methylation information of both CpG rich and poor regions.

In contrast, the density plots of β values for other normalisation (SWAN and FunNorm) methods do not improve to the same degree and in some cases greater variation is introduced (Additional file 3: Figure S2C–G). For example, a worsening of the batch effect is seen for SWAN normalisation (Additional file 3: Figure S2D), compared to raw data (Additional file 2: Figure S1A) and the distribution of methylated and unmethylated signals is inverted following FunNorm (Additional file 3: Figure S2E).

The second approach employed to examine the performance of the normalisation methods was to generate multidimensional scaling (MDS) plots. These permitted the visualisation of the two-dimensional projection of the differences between samples. For each plot, the 1000 most variable probes were selected, as these represent the most pertinent biological differences between samples. *M* values were used as opposed to β values, the latter of which have been shown to suffer severe heteroskedasticity at very high and low values [18]. Again, a strong batch effect is observed in the raw data (Fig. 2a) as expected and this is removed or significantly reduced following normalisation using stratified QN (Fig. 2c) and ComBat (Fig. 2e) corrected data. The strong batch effect masks the familial relationships in the raw data; however, following the correction, clustering according to kinship is clearly evident. Similarly, the replicate samples (in Additional file 2: Figure S1), which group disparately in the raw data (A, B), co-locate or cluster tightly following stratified QN (C, D) and ComBat (E, F). The MDS plots for each normalisation method (Additional file 4: Figure S3) also show stratified QN followed by ComBat to be the most effective method for removing clustering by batch.

This efficacy of normalisation methods in reducing clustering of samples by batch was assessed quantitatively by ANOVA to test the effect of batch on the first principal component. The ANOVA was repeated for each normalisation method, using *M* values from the top 1000 most variable sites. Consistent with the visualised MDS plot, the *p* value was highly significant demonstrating the significant association of batch in *M* value in raw and stratified QN data (*p* < 0.01) but was not significant following correction using ComBat (*p* = 0.97).

For a final qualitative measure to examine effectiveness of between array normalisation, hierarchical cluster dendrograms were generated. Application of stratified QN and ComBat (Additional file 5: Figure S4) again demonstrated superior normalisation when visualised by this method; with raw data samples clearly clustering into three distinct groups (Additional file 5: Figure S4A), stratified QN resulting in improved clustering (B) while ComBat batch correction following stratified QN completely removes the batch effect (C) permitting the desired outcome with related individuals clustering together in familial groups. Furthermore, replicate samples cluster more clearly after ComBat normalisation (C, red stars) indicating removal of batch effects without perturbing biologically relevant information.

To quantitatively assess the performance of these normalisation methods, the median absolute difference in *M* values was calculated for six replicate pairs, with one sample from each pair interrogated on a separate batch. With the exception of one pair, stratified QN with ComBat was found to have the lowest absolute median difference between technical replicate pairs, corresponding to the highest correlation between replicate pairs (see Additional file 6: Table S2). While others such as SWAN introduced an increase in the error rate relative to the raw data values.

Finally, standard error measures for imprinted regions were calculated and compared between methods as described in the statistical analysis section of the methods. Smaller values indicate lower errors and more reliable data. A DMRSE of 0.0048 was calculated for the raw data, with this value increasing with following normalisations using QN (0.0052), noob (0.0052) and functional normalisation (0.0056). The remaining normalisation methods generated reduced DMSRE values with stratified QN with ComBat batch correction again producing the smallest error values at 0.0012. See Additional file 7: Table S3 for a full list of DMRSE values and Additional file 8: Figure S5 for the density plots of these probes.

### Increased power for determining true biological associations

**Fig. 4:**
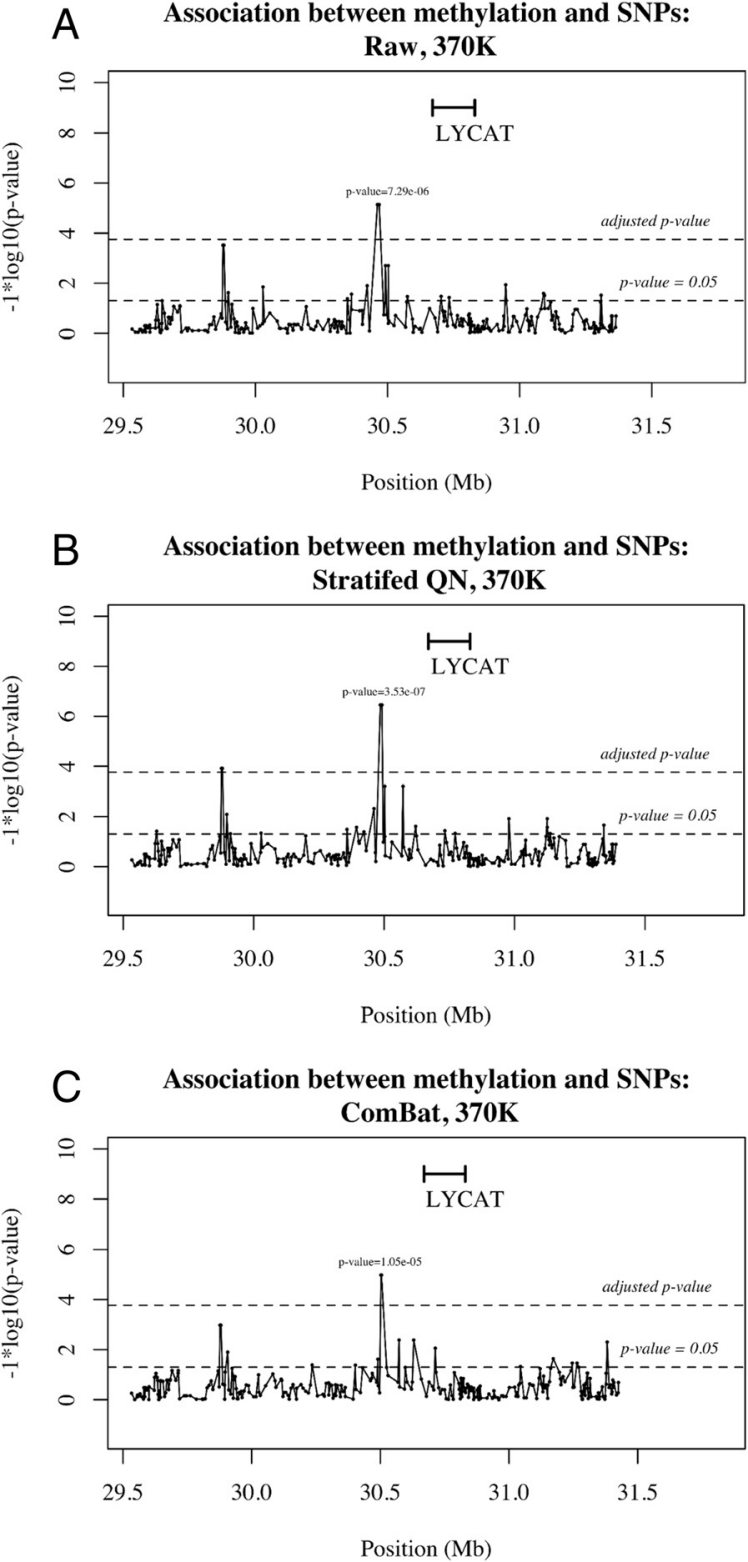
Association plot between SNPs and methylation. Association between methylation at cg17749961 and SNPs in a 2-Mb window. There is a significant association in the raw data (**a**, *p* value = 7.29e-06) which increases after stratified QN (**b**, *p* value = 3.53e-07) and drops slightly after ComBat correction (**c**, *p* value = 1.05e-05)

Critical to any normalisation method is the maintenance of true biological differences between samples. As described in the statistical analysis section of the methods, a previously identified meQTL was selected to perform association analysis with prior to and following normalisation. Following Bonferroni correction, a significant association was detected in the raw data (Fig. 4a, *p* value = 7.29e-06), increasing markedly after stratified QN (Fig. 4b, *p* value = 3.53e-07). After ComBat (C), there was a drop in significance compared to stratified QN and raw, yet the *p* value was still highly significant (*p* value = 1.05e-05) indicating preservation of the biological information of interest. The drop in significance after batch correction may be explained as confounding between batch and family, which is removed after ComBat. Ideally, samples would be randomised across experiments; however, the nature of familial studies is such that this is not always possible, as samples are collected at different time points, often across generations. To maintain maximum power, the inclusion of all available samples is essential and, therefore, data processing methods capable of dealing with non-ideal datasets are required.

Epigenome-wide methylation has long been shown to drift with age, specifically global hypomethylation and region-specific hypermethylation are observed [23]. The association between age and epigenome-wide methylation was compared for raw data, stratified QN normalised data and ComBat-corrected stratified QN data to demonstrate that this biological information was preserved after normalisation and batch correction. After normalisation (Additional file 9: Figure S6B), there are many more significant associations with age than in the raw data (Additional file 9: Figure S6A), indicated by a greater number of points above the expected line and a much greater Lambda value (median of observed −log10 *p* values divided by the median of expected −log10 *p* values), with an increase from 0.838 to 1.402. There is another small increase in significance after ComBat batch correction (Additional file 9: Figure S6C) to 1.448, again indicating improved strength in testing biological associations.

## Discussion

**Fig. 5:**
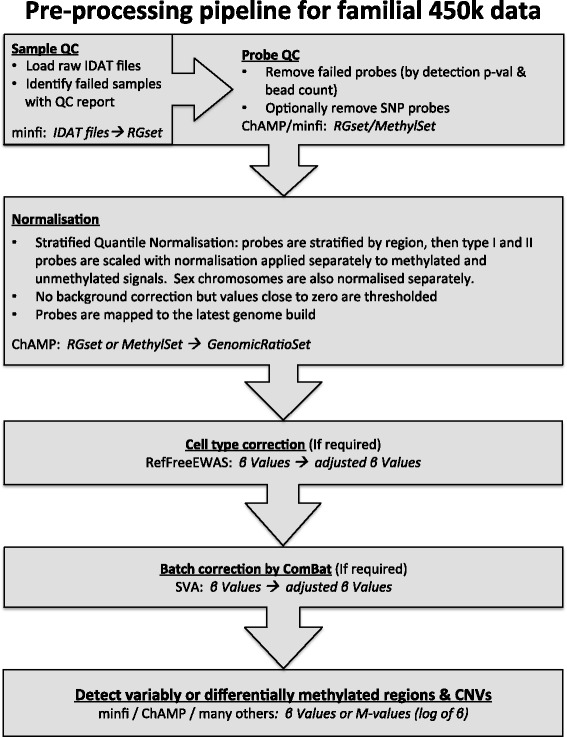
Pipeline for familial data processed on the 450k array. Each *box* indicates a stage of the pipeline including the R package and the data format required/created in *italics*

There is currently a plethora of pre-processing methods and R packages available for analysis of 450k array data, and comprehensive review articles evaluating their utility have been published [24–26]. The majority of these are designed for specific types of sample sets, particularly those comprised of two distinct groups such as case–control or cancer-normal with substantial methylation differences between the two groups. For different datasets, such as those from familial studies, which include complex pedigree structures instead of two distinct groups, these methods may be ineffective or worse, detrimental in that they introduce technical bias, as identified with selected methods in this paper. To correctly normalise data, it is critical to choose the most appropriate method; yet there has been little focus on developing appropriate processing pipelines for familial methylation array analysis, despite the current interest in inherited drivers of methylation patterns. Further barriers are the various format requirements and the lack of integration to provide a seamless processing pipeline. Here, we have tested eight different methods and presented a preliminary pre-processing pipeline for familial data (depicted in Fig. 5). This pipeline creates a template to guide and expedite the analysis of familial datasets, particularly generated using the 450k array data. Sample size (*n* = 50) is a limitation of this study, therefore additional familial studies would aid in validating the pipeline.

A fundamental requirement for processing methylation array data is effective adjustment for technical bias, including batch effects and adjusting for the two-probe biochemistry of the array. Batch effects may be introduced through bisulphite conversion or downstream processing or variation in array quality. Various methods have been developed to adjust for these effects, mostly involving variations in quantile normalisation, a technique commonly used in analysis of microarray datasets to align two different distributions so they result in identical statistical properties [26–29].

BMIQ and functional normalisation have been advocated as the preferred methods for cancer studies as they are more specific in design than quantile normalisation and have been shown to be more effective at removing unwanted technical bias [27, 29]. However, these methods work most effectively on case–control or tumour-normal datasets respectively and to the best of our knowledge, optimal pre-processing methods for familial-based data, such as performed here, have not been reported. Normalisation methods necessarily make assumptions about data, with the accuracy of these assumptions varying for different datasets. Thus, the same normalisation method can have a vastly different effect on different types of data and conversely, as shown here, different normalisation methods can have vastly different effects on the same data. It is therefore a key to select the right normalisation method for the dataset of interest. Of the eight methods tested, stratified QN was consistently identified as the best normalisation method across all visual and quantitative evaluation metrics for use in this context. The principle underpinning this normalisation is stratification by genomic region and is thus ideal for data where the differences between adjacent genomic loci are maintained. This is in contrast to tumour-normal tissue datasets where there are large blocks of dramatically altered methylation patterns throughout the tumour genome [30]. Again not surprisingly, packages that utilise differences in negative control methylation patterns between cases and controls such as FunNorm were not found to be effective on familial datasets where no ‘normal’ control is available.

The inherent strengths of familial data could be further exploited by a normalisation technique that accounts for known relationships between samples. Such a method could draw on pedigree information to ensure normalisation has effectively removed technical bias while maintaining known biologically relevant information such as relatedness and familial clustering by methylation. A diagnostic metric accounting for a known relationship could be used to test the efficacy of pre-processing methods in a similar manner to the standard error associated with iDMRs from the *wateRmelon* package.

It may also be of importance for researchers to consider the undesirable effect of non-specific binding and the presence of SNPs in the probe body. A study from the Weksberg lab found around 6 % of probes on the array cross-hybridised to non-targeted genomic regions [31]. They have catalogued these probes and suggest removing them prior to downstream analysis. Their study also demonstrates that SNPs in the probe body can interfere with probe binding, altering the methylation signal at around 14 % of sites. Illumina recommends all probes containing a SNP within 10 bp of the interrogated CpG site ought to be removed, while others suggest the ‘probe effect’ continues to the entire 50-bp length of the probe [31, 32]. The removal of all such probes would be undesirable for studies examining the effect of genotype on methylation, as evidence suggests the vast majority of these SNPs occur either at the CpG site itself (meSNPs) or close by [32].

To overcome this issue, Zhi and colleagues suggest an elegant approach to examine the effect of meSNPs on methylation without the potential bias introduced by SNPs altering probe binding [32]. The type II probes contain only one bead type for both methylated and unmethylated sites of interest, with the methylation status of the loci designated by the addition of a different coloured nucleotide (red or green) at the single base extension. As type II probes terminate one base pair before the cytosine of the CpG dinucleotide, a mutation at the cytosine itself would not affect probe binding. As such, probes without SNPs in the probe body but present at the single base extension can reliably be used to examine the effect of meSNPs on methylation, a very useful technique for examining the effect of inherited variation on methylation patterns.

## Conclusions

Preservation of the biological integrity of information from methylation array data is imperative and requires appropriate pre-processing to minimise technical errors, which will be dictated by the type of data. Stratified QN in combination with ComBat batch correction performed the best of those methods tested for normalising familial data interrogated on 450k array. This method was observed to remove technical biases while maintaining biologically relevant information; allowing true biological differences and similarities to inform our search for the role of methylation patterns driving disease processes. The workflow presented in this paper (highlighted in Fig. 5) provides a streamlined methodology to pre-process familial data and may also be instructive for other datasets including longitudinal studies where the same individuals are repeatedly measured over time.

## Abbreviations

CpG, cytosine-guanine pair; meQTL, methylation quantitative trait loci; MDS, multidimensional scaling; meSNPs, methylation single nucleotide polymorphisms

## Additional files

Additional file 1: Table S1. Clinical data and sample extraction and storage information. (DOCX 20 kb) Additional file 2: Figure S1. Hierarchical cluster dendrogram for raw, stratified QN and ComBat-corrected data. Samples are clustered by similarity and labelled by batch. Raw data samples (A) clearly cluster into three distinct batches while stratified QN (B) partially adjusts clustering by batch and stratified QN combined with ComBat considerably diminishes the batch effect (C). Red stars indicate replicate samples which cluster more clearly in (C), indicating removal of batch effects. (PDF 449 kb) Additional file 3: Figure S2. Density distribution of β values and multidimensional scaling plots of *M* values for replicate samples. Density (A, C, E) and MDS (B, D, F) plots of three replicate sample groups for raw (A, B), stratified QN (C, D) and stratified QN ComBat-corrected (E, F) data. For all plots, samples are coloured by batch 1–3 as labelled. Density plots show the distribution of β values, which become more uniform after stratified QN (C) and stratified QN plus ComBat (E). MDS plots show clustering of the 1000 most variable sites by *M* value, highlighting the decreasing variance between replicate groups after stratified QN and ComBat (F). (PDF 7387 kb) Additional file 4: Figure S3. Density distribution of β values for imprinted differentially methylated regions. Density plots for raw (A), stratified QN (C) and stratified QN with ComBat (E) for 227 probes mapping known imprinted differentially methylated regions. Each line represents a sample, with samples coloured by batch. As methylation at these loci is allele-specific there is a single density distribution rather than the bimodal distribution seen in Additional file 3: Figure S2. The standard error-type measure (DMRSE) diminishes with Stratified QN and ComBat, indicating more reliable data. B, D and F show the Infinium I and II probe distributions, which becomes more uniform with stratified QN and ComBat. (PDF 4133 kb) Additional file 5: Figure S4. Density distribution of β values for all normalisation methods. Density plots of β values for various normalisation methods: raw pre-normalisation data (A), quantile normalisation (B), BMIQ (C), SWAN (D), FunNorm (E), Dasen (F), noob (G), stratified QN (H), raw with ComBat correction (I) and stratified QN with ComBat correction (J). A single line represents a sample with samples coloured by batch. The batch effect present in the raw data (A) remains after the majority of normalisation methods with Dasen (F) and stratified QN (H) showing the most uniform distributions. Some methods such as quantile normalisation (B) and FunNorm (E) flip the methylated and unmethylated signal distribution. ComBat is effective at removing batch effects in both raw (I) and normalised (J) data, with the best outcome seen with stratified QN with ComBat batch correction (J). (PDF 260 kb) Additional file 6: Table S2. Median absolute difference between technical replicate pairs. (DOCX 14 kb) Additional file 7: Table S3. Standard error measures for imprinted differentially methylated regions for the various normalisation methods. (DOCX 13 kb) Additional file 8: Figure S5. Multidimensional scaling plots of *M* values by batch for all normalisation methods. Multidimensional scaling plots for raw (A), quantile normalisation (B), BMIQ (C), SWAN (D), FunNorm (E), Dasen (F), noob (G), stratified QN (H), raw with ComBat correction (I) and stratified QN with ComBat correction (J). For each plot, the 1000 most variable probes were selected. Batches are numbered and coloured, with clustering by batch clearly seen in the raw data (A) and removed to varying degrees with different normalisation methods. ComBat correction following stratified QN provides optimal batch correction removal as the samples no longer cluster according to batch. (PDF 559 kb) Additional file 9: Figure S6. QQ plots for the association of age and epigenome-wide methylation. QQ plots with −log10 *p* values from the linear model of methylation and age plotted against expected −log10 *p* values. Raw data (A), data normalised by stratified QN (B) and data normalised by stratified QN then corrected with ComBat (C). (PDF 85 kb)
