# Supplementary material for: Comparison of pre-processing methodologies for Illumina 450k methylation array data in familial analyses
Source: Clin Epigenetics. 2016 Jul 16;8:75. doi: 10.1186/s13148-016-0241-2 (PMC4947255; doi:10.1186/s13148-016-0241-2)
Supplement: Additional file 1: Table S1. — Clinical data and sample extraction and storage information. (DOCX 20 kb) [file 13148_2016_241_MOESM1_ESM.docx]

**Table S1. Clinical data and sample extraction and storage information.**

| **SAMPLE**  **ID** | **SEX** | **STATUS** | **STORAGE** | **DATE**  **COLLECTED** | **DATE**  **EXTRACTED** | **AGE AT**  **COLLECTION** |
| --- | --- | --- | --- | --- | --- | --- |
| PC11-03 | Male | Affected | 4° | NA | NA | 89 |
| PC11-04 | Male | Affected | 4° | NA | NA | 73 |
| PC11-09 | Male | Affected | 4° | 26.03.2004 | 27.04.2004 | 83 |
| PC11-147 | Male | Affected | 4° | 05.11.2004 | 12.11.2004 | 61 |
| PC11-180 | Male | Unaffected | 4° | 20.11.2004 | 29.11.2004 | 42 |
| PC11-213 | Male | Affected | 4° | 18.07.2005 | 06.09.2005 | 62 |
| PC11-233 | Male | Unaffected | 4° | 12.10.2005 | 08.11.2005 | 92 |
| PC11-234 | Male | Unaffected | 4° | 14.08.2004 | 19.08.2004 | 55 |
| PC11-415 | Male | Unaffected | 4° | 12.10.2005 | 08.11.2005 | 61 |
| PC22-02 | Male | Affected | 4° | NA | NA | 64 |
| PC22-03 | Male | Affected | 4° | NA | NA | 74 |
| PC22-04 | Male | Affected | 4° | NA | NA | 62 |
| PC22-16 | Male | Affected | 4° | 26.03.2004 | 07.04.2004 | 76 |
| PC22-162 | Male | Unaffected | 4° | 06.02.2004 | 07.04.2004 | 56 |
| PC22-17 | Male | Affected | 4° | NA | NA | 63 |
| PC22-195 | Female | NA | 4° | 06.04.2005 | 13.04.2005 | 40 |
| PC22-203 | Male | Affected | 4° | 26.08.2004 | 27.09.2004 | 75 |
| PC22-21 | Male | Affected | 4° | 22.04.2004 | 30.04.2004 | 70 |
| PC22-210 | Female | NA | 4° | 07.12.2004 | 14.12.2004 | 73 |
| PC22-274 | Male | Unaffected | 4° | 06.04.2005 | 13.04.2005 | 45 |
| PC22-386 | Female | NA | 4° | 08.10.2004 | 25.10.2004 | 56 |
| PC22-387 | Male | Affected | 4° | 30.09.2004 | 12.10.2004 | 79 |
| PC22-388 | Male | Unaffected | 4° | 19.11.2004 | 29.11.2004 | 73 |
| PC22-393 | Female | NA | 4° | 10.10.2004 | 25.10.2004 | 44 |
| PC22-414 | Female | NA | 4° | 09.10.2004 | 25.10.2004 | 66 |
| PC22-416 | Male | Affected | 4° | 10.10.2004 | 25.10.2004 | 61 |
| PC22-418 | Male | Unaffected | 4° | 09.10.2004 | 25.10.2004 | 54 |
| PC22-468 | Male | Affected | 4° | 09.10.2004 | 25.10.2004 | 69 |
| PC22-476 | Male | Unaffected | 4° | 11.12.2004 | 20.12.2004 | 36 |
| PC72-03 | Male | Affected | 4° | 21.10.1998 | NA | 70 |
| PC72-04 | Male | Affected | 4° | NA | NA | 78 |
| PC72-106 | Male | Unaffected | 4° | 10.08.2004 | 20.08.2004 | 46 |
| PC72-126 | Male | Unaffected | 4° | 10.10.2004 | 25.10.2004 | 49 |
| PC72-136 | Male | Other Cancer | 4° | 24.08.2004 | 26.08.2004 | 57 |
| PC72-187 | Female | NA | 4° | 15.10.2004 | 29.10.2004 | 41 |
| PC72-188 | Male | Other Cancer | 4° | 06.04.2005 | 13.04.2005 | 23 |
| PC72-213 | Male | Unaffected | 4° | 22.03.2005 | 12.04.2005 | 41 |
| PC72-291 | Male | Unaffected | 4° | 24.05.2005 | 08.11.2005 | 42 |
| PC72-77 | Male | Affected | 4° | 02.03.2004 | 28.04.2004 | 75 |
| PC9-01 | Male | Affected | 4° | NA | NA | 64 |
| PC9-04 | Male | Affected | 4° | NA | NA | 65 |
| PC9-12 | Male | Affected | 4° | 04.12.2003 | NA | 72 |
| PC9-121 | Male | Unaffected | 4° | 29.01.2004 | NA | 48 |
| PC9-129 | Female | NA | 4° | 28.01.2004 | 27.04.2004 | 61 |
| PC9-24 | Female | NA | 4° | 22.01.2004 | NA | 45 |
| PC9-286 | Male | Unaffected | 4° | 06.10.2004 | 13.10.2004 | 47 |
| PC9-29 | Female | NA | 4° | 29.01.2004 | 27.04.2004 | 71 |
| PC9-338 | Male | Affected | 4° | 10.12.2004 | 20.12.2004 | 63 |
| PC9-357 | Male | Unaffected | 4° | 07.04.2005 | 13.04.2005 | 42 |
| PC9-477 | Male | Affected | 4° | 08.10.2004 | 18.10.2004 | 52 |
