# Supplementary material for: Comparison of pre-processing methodologies for Illumina 450k methylation array data in familial analyses
Source: Clin Epigenetics. 2016 Jul 16;8:75. doi: 10.1186/s13148-016-0241-2 (PMC4947255; doi:10.1186/s13148-016-0241-2)
Supplement: Additional file 2: Figure S1. — Hierarchical cluster dendrogram for raw, stratified QN and ComBat-corrected data. Samples are clustered by similarity and labelled by batch. Raw data samples (A) clearly cluster into three distinct batches while stratified QN (B) partially adjusts clustering by batch and stratified QN combined with ComBat considerably diminishes the batch effect (C). Red stars indicate replicate samples which cluster more clearly in (C), indicating removal of batch effects. (PDF 449 kb) [file 13148_2016_241_MOESM2_ESM.pdf]

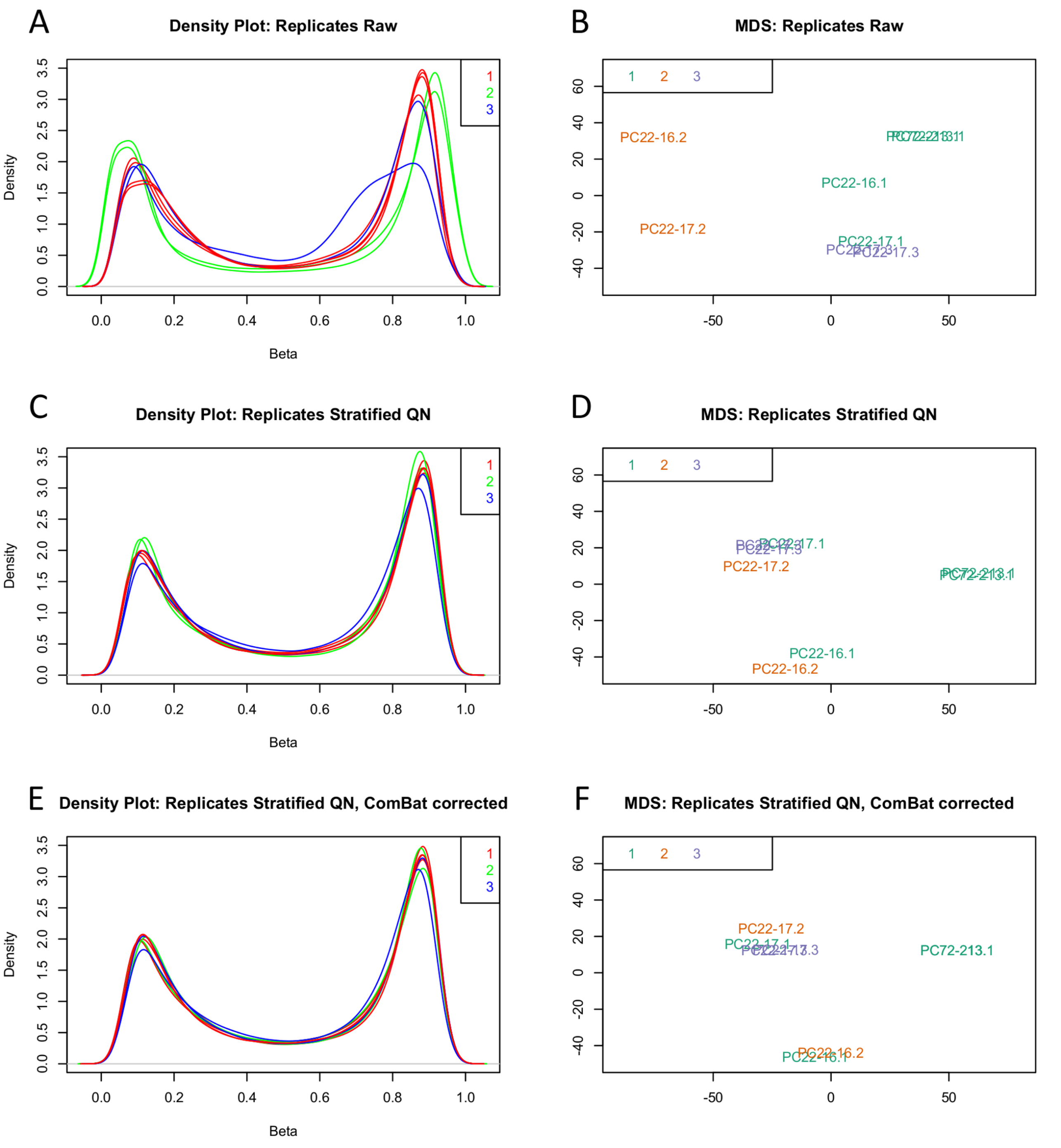
A
Density Plot: Replicates Raw
Density
Beta
0.0
0.2
0.4
0.6
0.8
1.0
0.0
0.5
1.0
1.5
2.0
2.5
3.0
3.5
1
2
3
B
MDS: Replicates Raw
1
2
3
PC22-16.2
PC22-16.1
PC22-17.2
PC22-17.1
PC22-17.3
-50
0
50
60
40
20
0
-20
-40
C
Density Plot: Replicates Stratified QN
Density
Beta
1
2
3
D
MDS: Replicates Stratified QN
1
2
3
PC22-17.1
PC22-17.3
PC22-17.2
PC72-213.1
PC22-16.1
PC22-16.2
E
Density Plot: Replicates Stratified QN, ComBat corrected
Density
Beta
1
2
3
F
MDS: Replicates Stratified QN, ComBat corrected
1
2
3
PC22-17.2
PC22-17.1
PC72-213.1
PC22-16.2
PC22-16.1
