# Supplementary figures and images for: Comparison of pre-processing methodologies for Illumina 450k methylation array data in familial analyses
Source: Clin Epigenetics. 2016 Jul 16;8:75. doi: 10.1186/s13148-016-0241-2 (PMC4947255; doi:10.1186/s13148-016-0241-2)

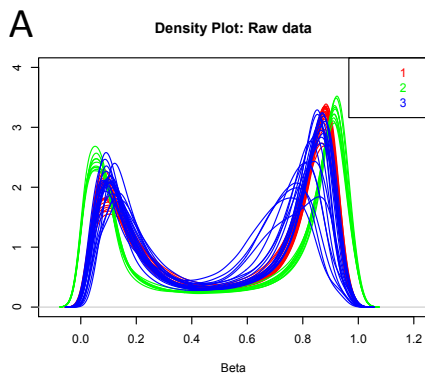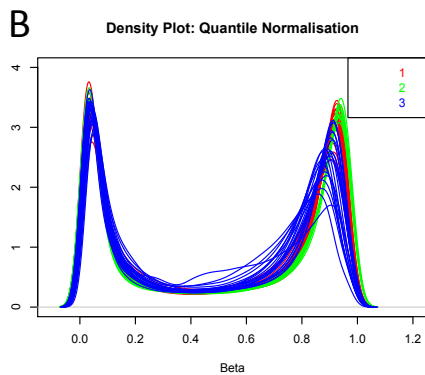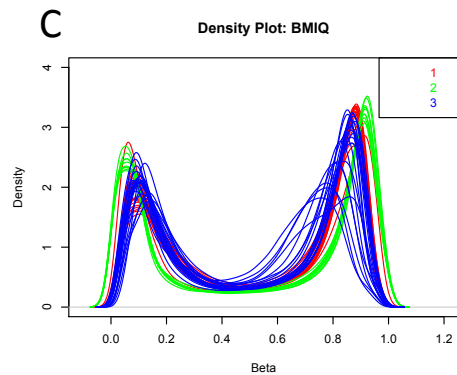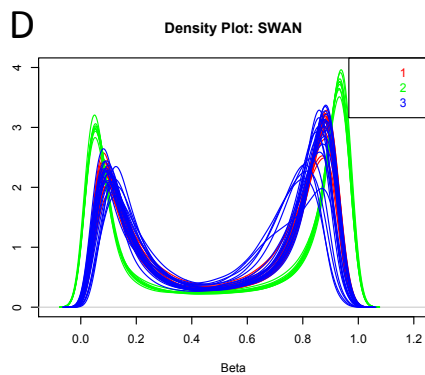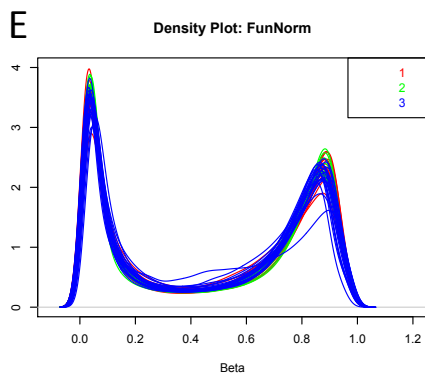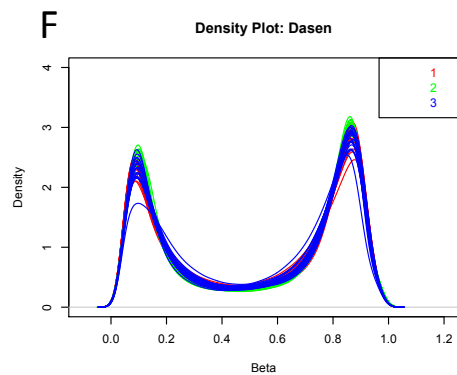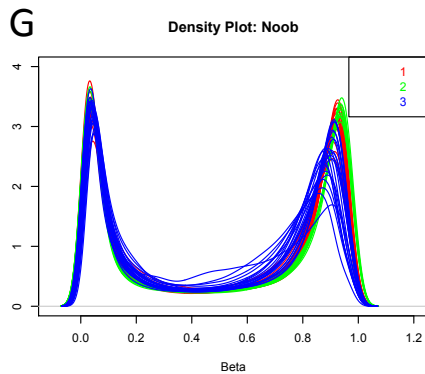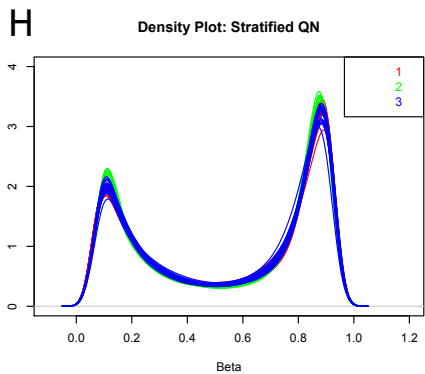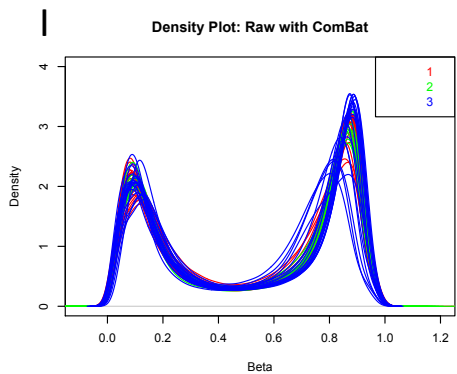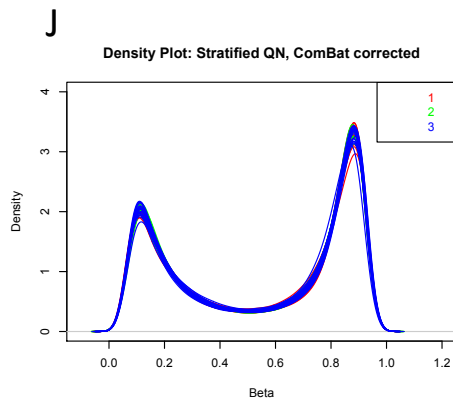

Supplement: Additional file 3: Figure S2. — Density distribution of β values and multidimensional scaling plots of M values for replicate samples. Density (A, C, E) and MDS (B, D, F) plots of three replicate sample groups for raw (A, B), stratified QN (C, D) and stratified QN ComBat-corrected (E, F) data. For all plots, samples are coloured by batch 1–3 as labelled. Density plots show the distribution of β values, which become more uniform after stratified QN (C) and stratified QN plus ComBat (E). MDS plots show clustering of the 1000 most variable sites by M value, highlighting the decreasing variance between replicate groups after stratified QN and ComBat (F). (PDF 7387 kb) [file 13148_2016_241_MOESM3_ESM.pdf]

A

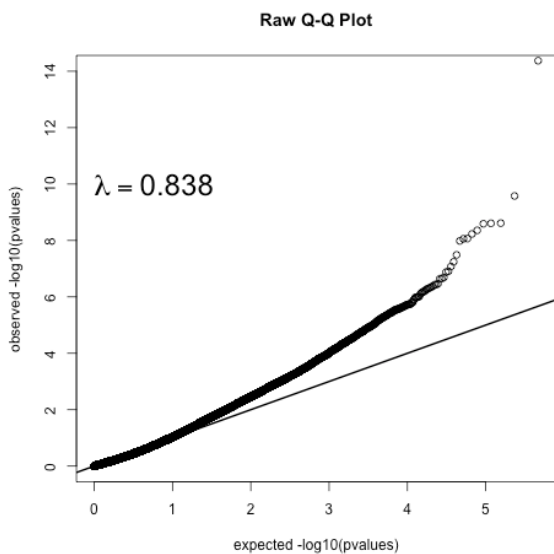

B

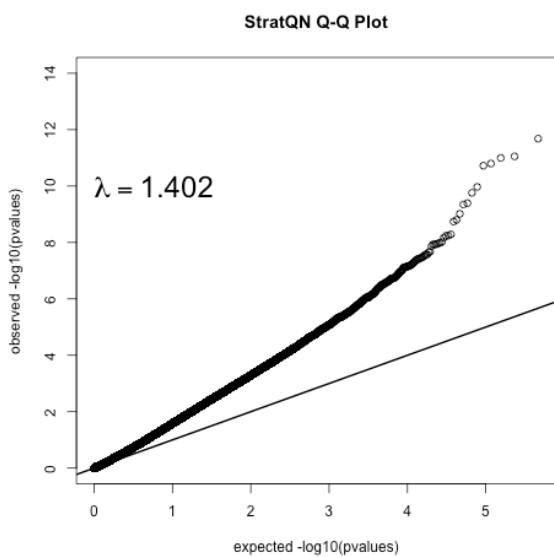

C

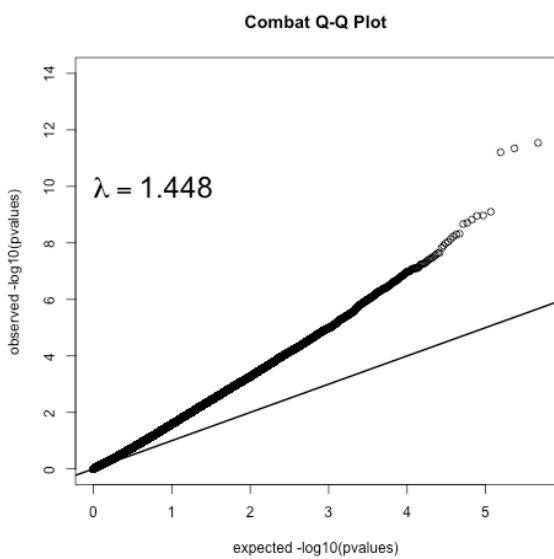

Supplement: Additional file 9: Figure S6. — QQ plots for the association of age and epigenome-wide methylation. QQ plots with −log10 p values from the linear model of methylation and age plotted against expected −log10 p values. Raw data (A), data normalised by stratified QN (B) and data normalised by stratified QN then corrected with ComBat (C). (PDF 85 kb) [file 13148_2016_241_MOESM9_ESM.pdf]
