# Supplementary material for: Comparison of pre-processing methodologies for Illumina 450k methylation array data in familial analyses
Source: Clin Epigenetics. 2016 Jul 16;8:75. doi: 10.1186/s13148-016-0241-2 (PMC4947255; doi:10.1186/s13148-016-0241-2)
Supplement: Additional file 4: Figure S3. — Density distribution of β values for imprinted differentially methylated regions. Density plots for raw (A), stratified QN (C) and stratified QN with ComBat (E) for 227 probes mapping known imprinted differentially methylated regions. Each line represents a sample, with samples coloured by batch. As methylation at these loci is allele-specific there is a single density distribution rather than the bimodal distribution seen in Additional file 3: Figure S2. The standard error-type measure (DMRSE) diminishes with Stratified QN and ComBat, indicating more reliable data. B, D and F show the Infinium I and II probe distributions, which becomes more uniform with stratified QN and ComBat. (PDF 4133 kb) [file 13148_2016_241_MOESM4_ESM.pdf]

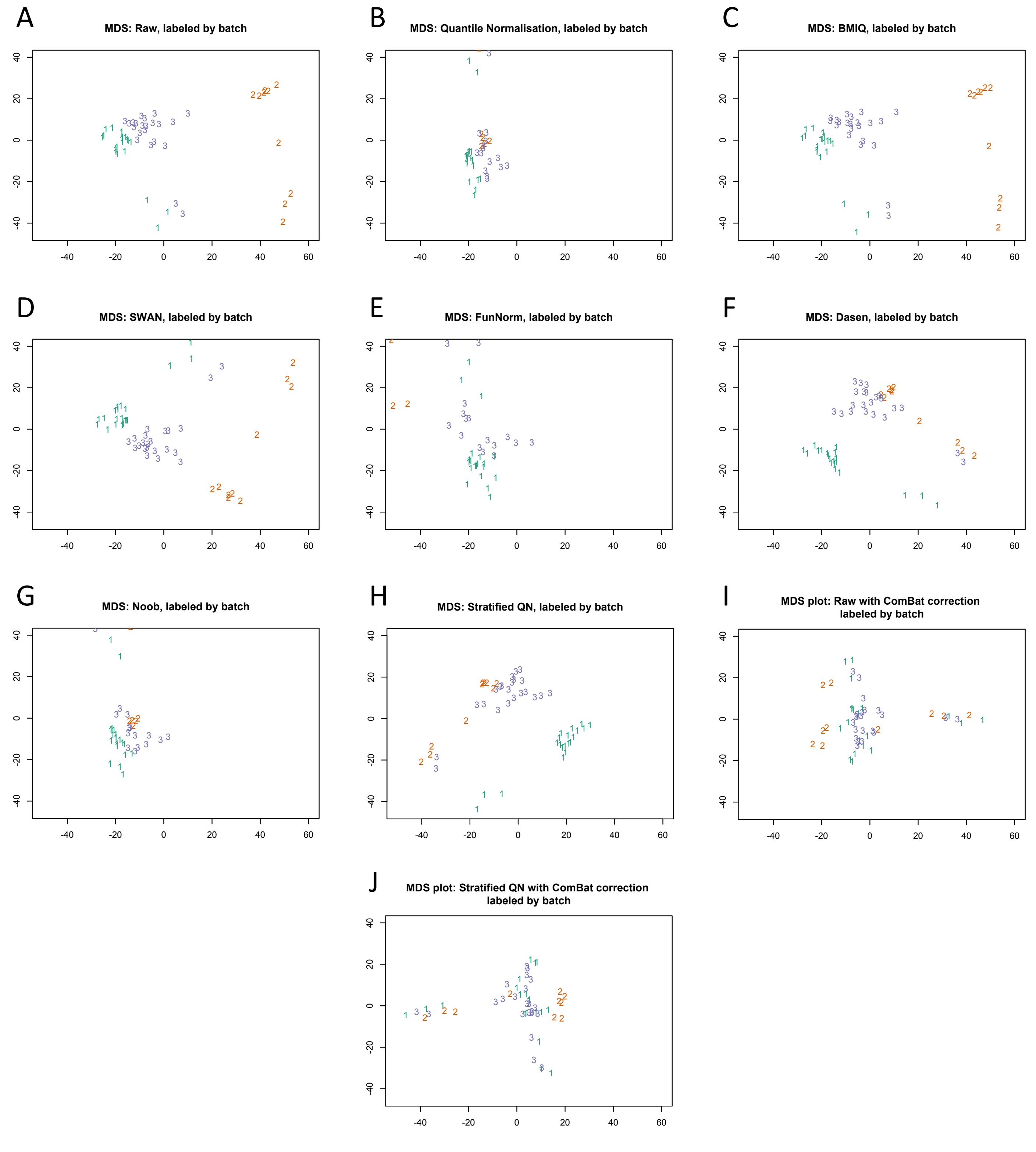
A
MDS: Raw, labeled by batch
B
MDS: Quantile Normalisation, labeled by batch
C
MDS: BMIQ, labeled by batch
D
MDS: SWAN, labeled by batch
E
MDS: FunNorm, labeled by batch
F
MDS: Dasen, labeled by batch
G
MDS: Noob, labeled by batch
H
MDS: Stratified QN, labeled by batch
I
MDS plot: Raw with ComBat correction labeled by batch
J
MDS plot: Stratified QN with ComBat correction labeled by batch
