# Supplementary material for: Comparison of pre-processing methodologies for Illumina 450k methylation array data in familial analyses
Source: Clin Epigenetics. 2016 Jul 16;8:75. doi: 10.1186/s13148-016-0241-2 (PMC4947255; doi:10.1186/s13148-016-0241-2)
Supplement: Additional file 5: Figure S4. — Density distribution of β values for all normalisation methods. Density plots of β values for various normalisation methods: raw pre-normalisation data (A), quantile normalisation (B), BMIQ (C), SWAN (D), FunNorm (E), Dasen (F), noob (G), stratified QN (H), raw with ComBat correction (I) and stratified QN with ComBat correction (J). A single line represents a sample with samples coloured by batch. The batch effect present in the raw data (A) remains after the majority of normalisation methods with Dasen (F) and stratified QN (H) showing the most uniform distributions. Some methods such as quantile normalisation (B) and FunNorm (E) flip the methylated and unmethylated signal distribution. ComBat is effective at removing batch effects in both raw (I) and normalised (J) data, with the best outcome seen with stratified QN with ComBat batch correction (J). (PDF 260 kb) [file 13148_2016_241_MOESM5_ESM.pdf]

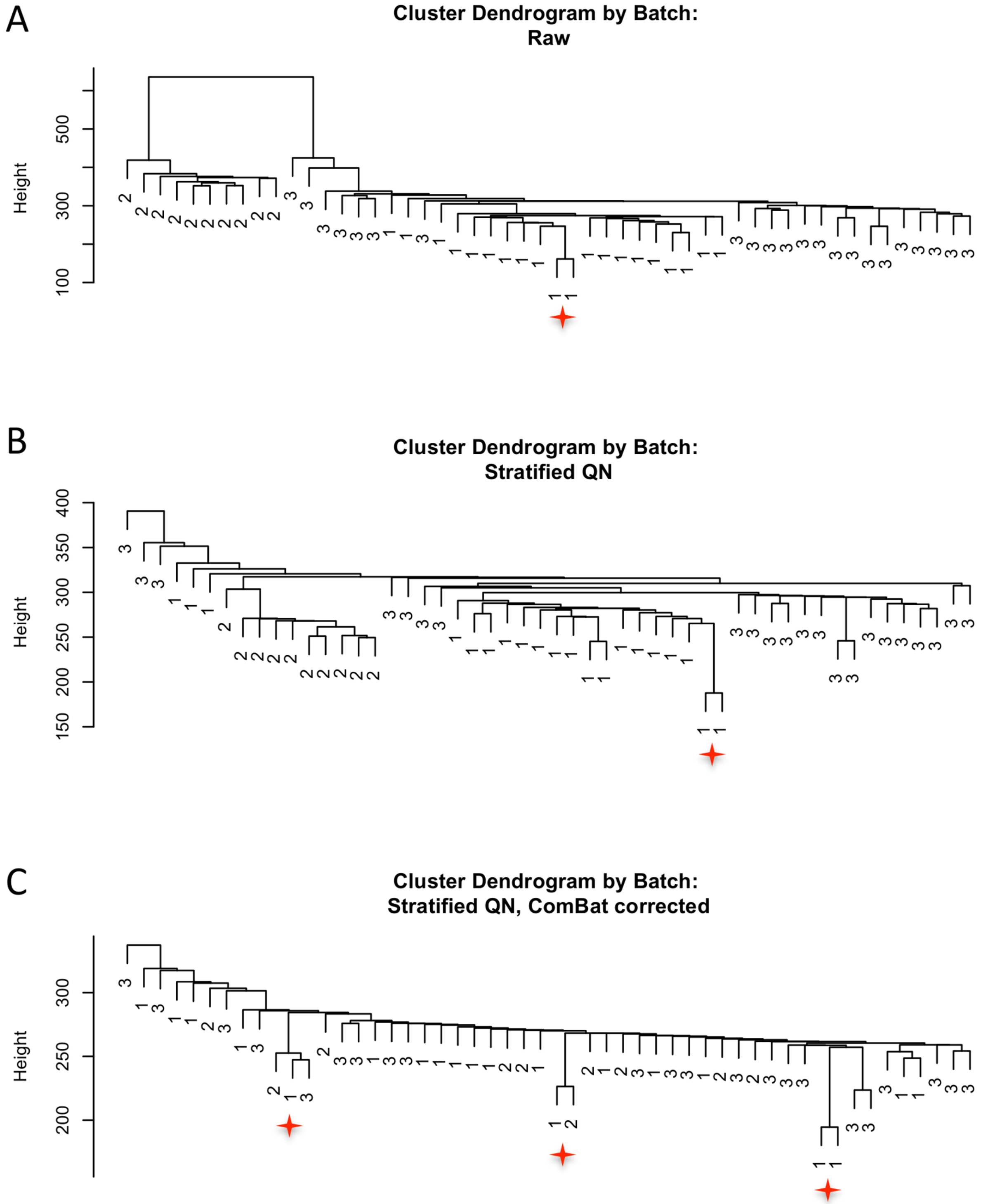
A
Cluster Dendrogram by Batch:
Raw
Height
100
300
500
B
Cluster Dendrogram by Batch:
Stratified QN
Height
150
200
250
300
350
400
C
Cluster Dendrogram by Batch:
Stratified QN, ComBat corrected
Height
200
250
300
