# Supplementary material for: Comparison of pre-processing methodologies for Illumina 450k methylation array data in familial analyses
Source: Clin Epigenetics. 2016 Jul 16;8:75. doi: 10.1186/s13148-016-0241-2 (PMC4947255; doi:10.1186/s13148-016-0241-2)
Supplement: Additional file 6: Table S2. — Median absolute difference between technical replicate pairs. (DOCX 14 kb) [file 13148_2016_241_MOESM6_ESM.docx]

**Table S2. Median absolute difference between technical replicate pairs.**

| **Normalisation Method** | **Pair.1** | **Pair.2** | **Pair.3** | **Pair.4** | **Pair.5** | **Pair.6** |
| --- | --- | --- | --- | --- | --- | --- |
| Raw | 0.557 | 0.397 | 0.639 | 0.255 | 0.974 | 0.721 |
| Quantile Normalisation | 0.335 | 0.612 | 0.378 | 0.322 | 0.610 | 0.414 |
| Stratified Quantile Normalisation | 0.258 | 0.377 | 0.309 | 0.268 | 0.381 | 0.330 |
| BMIQ | 0.569 | 0.414 | 0.646 | 0.271 | 0.980 | 0.726 |
| SWAN | 0.676 | 0.375 | 0.751 | 0.247 | 1.003 | 0.808 |
| Functional Normalisation | 0.334 | 0.511 | 0.378 | 0.312 | 0.590 | 0.398 |
| Dasen | 0.250 | 0.399 | 0.290 | 0.253 | 0.399 | 0.313 |
| Noob | 0.414 | 0.646 | 0.410 | 0.411 | 0.916 | 0.621 |
| Raw with ComBat | 0.263 | 0.268 | 0.258 | 0.236 | 0.336 | 0.261 |
| Stratified Quantile Normalisation with ComBat | 0.193 | 0.313 | 0.218 | 0.210 | 0.270 | 0.223 |
