# Supplementary material for: Comparison of pre-processing methodologies for Illumina 450k methylation array data in familial analyses
Source: Clin Epigenetics. 2016 Jul 16;8:75. doi: 10.1186/s13148-016-0241-2 (PMC4947255; doi:10.1186/s13148-016-0241-2)
Supplement: Additional file 7: Table S3. — Standard error measures for imprinted differentially methylated regions for the various normalisation methods. (DOCX 13 kb) [file 13148_2016_241_MOESM7_ESM.docx]

**Table S3. Standard error measures for imprinted differentially methylated regions for the various normalisation methods.**

| **Normalisation Method** | **DMRSE** |
| --- | --- |
| Raw | 0.0048 |
| Quantile Normalisation | 0.0052 |
| Stratified Quantile Normalisation | 0.0028 |
| BMIQ | 0.0048 |
| SWAN | 0.0046 |
| Functional Normalisation | 0.0056 |
| Dasen | 0.0043 |
| Noob | 0.0052 |
| Raw with ComBat | 0.0028 |
| Stratified Quantile Normalisation + ComBat | 0.0012 |
