# Supplementary material for: Comparison of pre-processing methodologies for Illumina 450k methylation array data in familial analyses
Source: Clin Epigenetics. 2016 Jul 16;8:75. doi: 10.1186/s13148-016-0241-2 (PMC4947255; doi:10.1186/s13148-016-0241-2)
Supplement: Additional file 8: Figure S5. — Multidimensional scaling plots of M values by batch for all normalisation methods. Multidimensional scaling plots for raw (A), quantile normalisation (B), BMIQ (C), SWAN (D), FunNorm (E), Dasen (F), noob (G), stratified QN (H), raw with ComBat correction (I) and stratified QN with ComBat correction (J). For each plot, the 1000 most variable probes were selected. Batches are numbered and coloured, with clustering by batch clearly seen in the raw data (A) and removed to varying degrees with different normalisation methods. ComBat correction following stratified QN provides optimal batch correction removal as the samples no longer cluster according to batch. (PDF 559 kb) [file 13148_2016_241_MOESM8_ESM.pdf]

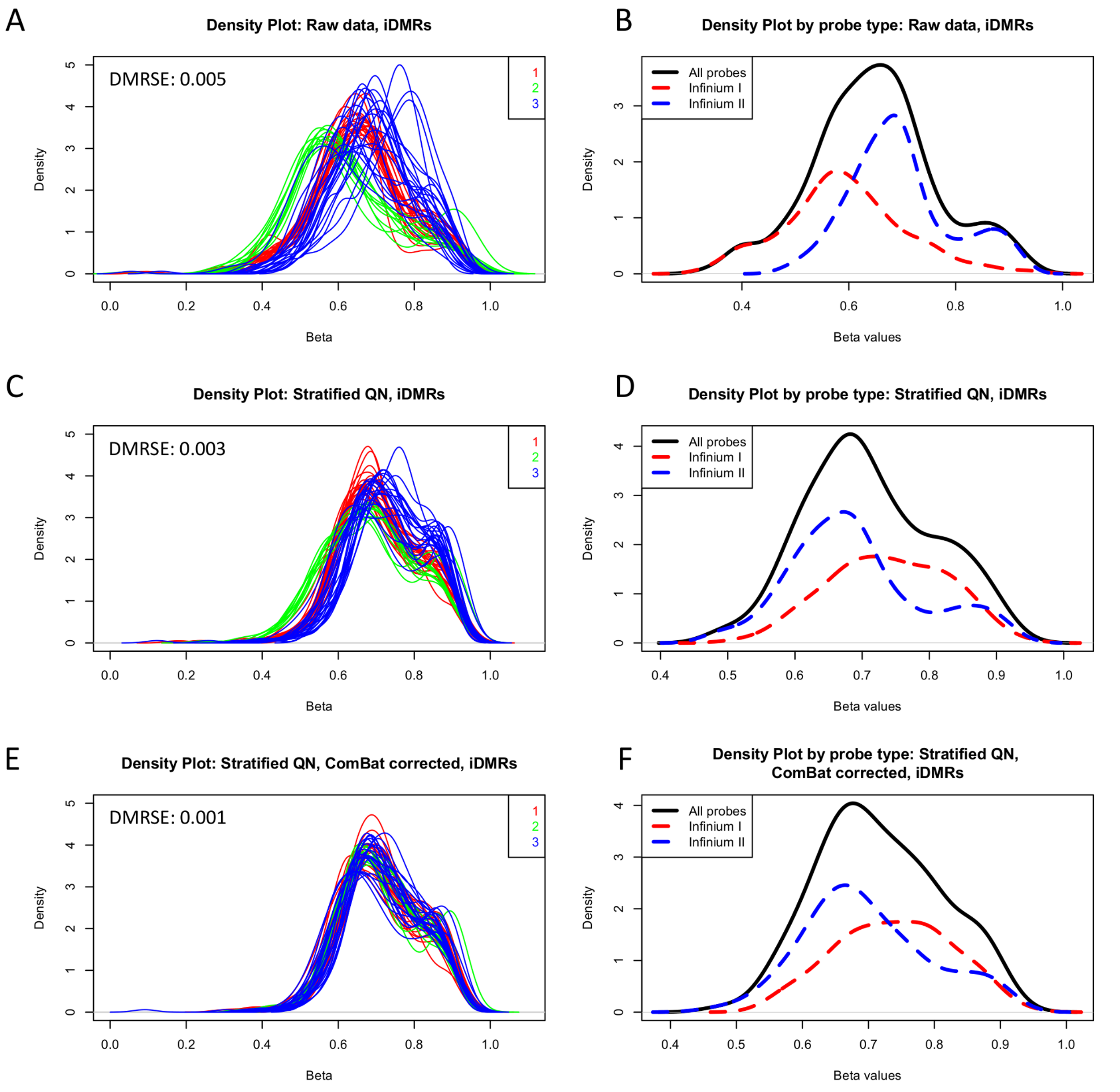
A
Density Plot: Raw data, iDMRs
DMRSE: 0.005
1
2
3
Density
Beta
B
Density Plot by probe type: Raw data, iDMRs
All probes
Infinium I
Infinium II
Density
Beta values
C
Density Plot: Stratified QN, iDMRs
DMRSE: 0.003
Density
Beta
D
Density Plot by probe type: Stratified QN, iDMRs
E
Density Plot: Stratified QN, ComBat corrected, iDMRs
DMRSE: 0.001
F
Density Plot by probe type: Stratified QN, ComBat corrected, iDMRs
